# Appraisal of groundwater conditions through hydrogeophysical and hydrogeological approach in Cholistan area, district Bahawalpur, Punjab, Pakistan

**DOI:** 10.1371/journal.pone.0317729

**Published:** 2025-05-07

**Authors:** Shahbaz Muhammad, Perveiz Khalid, Muhammad Irfan Ehsan, Umar Javed, Qazi Adnan Ahmad, Irfan Raza, Naser Golsanami, Shahzada Khurram

**Affiliations:** 1 College of Energy and Mining Engineering, Shandong University of Science and Technology, Qingdao, China; 2 Geological Survey of Pakistan, Lahore, Pakistan; 3 Institute of Geology, University of the Punjab, Lahore, Pakistan; 4 Department of Agricultural & Biosystems Engineering, South Dakota State University, Brookings, South Dakota, United States of America; 5 School of Mines, China University of Mining and Technology, Xuzhou, Jiangsu, China; National College Autonomous, INDIA

## Abstract

The sustainable groundwater supply has a prime importance for the development of an area. Unfortunately, the groundwater-related issues are heading towards terrible situation regionally and locally as well. As far as the different parts of Punjab Province of Pakistan are concerned, Cholistan is almost a desert region where the groundwater scarcity is being observed in its different parts since decades. The present research is an attempt to assess the latest groundwater potential and demarcation of different groundwater quality zones exist at subsurface in the study area of Cholistan. For this purpose, hydrogeophysical studies allied with hydrogeological approach were utilized. Hydrogeophysical investigations were carried out in the study area at evenly distributed 50 sites by deploying geo-electrical survey. Lithology represented by the boreholes and geo-electrical survey results altogether deduced that the eolian deposits overlie with intermixed fine and coarse alluvium. These sediments are mainly intermixed layers of sand, clay, silt, gravel and some kankars. Overall three to six subsurface geo-electric layers were identified through the geo-electrical survey and the resistivity values were categorized as high, medium and low across the study area. 2D and 3D maps were developed deciphering different groundwater quality zones in terms of electrical resistivity behaviour with depth. Spatial maps of hydrogeological values in terms of Dar-Zourrak parameters were also developed for further precise demarcation of different qualities of groundwater pockets. Furthermore, hydraulic parameters were estimated and represented in 2D maps to evaluate aquifer potential zones across the study area. Hydrogeophysical and hydrogeological approaches integrally depict that most of the part of study area holds better groundwater conditions with high potential but the salinity prevails in some parts that goes to persist with depth.

## Introduction

The sustainable availability of quality groundwater is essential for the socio-economic development of an area. The surface water resources are cheapest and easily available. However, unfortunately these resources are not sufficient which leads to the dependency of subsurface water commonly known as groundwater. Again unfortunately, the sustainability of fresh/good quality groundwater is rapidly decreasing regionally and locally as well. By the time we cross the threshold of year 2060, it is estimated that the two-third of world’s population could be living under water stressed conditions. Moreover, almost 4 billion people will be grappling with the water scarcity. Definitely, the culprits are the climate changes, global warming and overexploitation of the invisible groundwater reservoirs [1–3].

**Fig 1:**
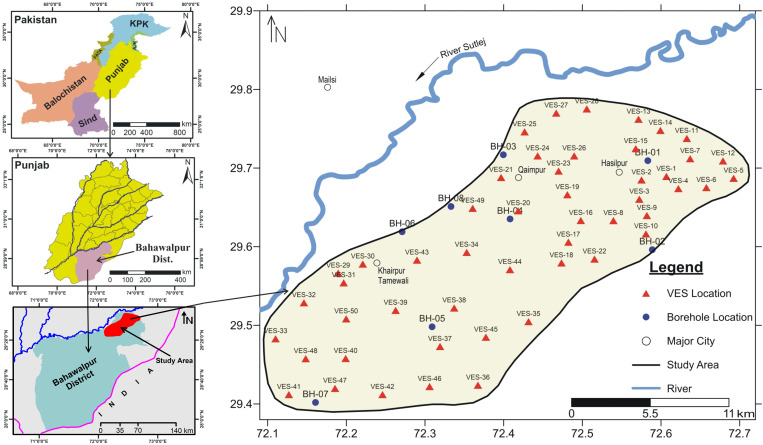
Study area map showing locations of boreholes and VES probes.

Pakistan is among those countries which are facing water scarcity. Currently, the country is 50% water supply deficient. In 1950s, the per capita water availability was 5000 m³, in 2010 it was decreased up to 1000 m³ and currently it is about 850 m³. Punjab Province of Pakistan; the land of five rivers is also facing water scarcity where the good quality shallow groundwater level has been dropped 27–19% in last decade [2–3]. The study area of the present research work is the part of Cholistan area (latitude 29.38°–29.8° and longitude 72.09°–72.72°). Politically the study area lies at northeast of dist. Bahawalpur in Punjab Province of Pakistan (Fig 1) covering the largest area among all districts of Punjab Province. Cholistan covers an area of about 25,000 km^2^ and extends into Thar Desert towards the south [4–6]. Almost the whole of Cholistan has been facing water shortage and salinity issues for centuries. The region was once watered by the Ghagra-Hakra River known as the Sarasvati in vedic times. The rainfall is minimal compared with the other parts of the province and little cultivation is made possible in the far-off desert parts by underground wells drawn up by the camels. Water is stored in troughs, built by the inhabitants, between sandhills and din waterholes called tobas [7, 8].

Cholistan desert is divided into three parts according to its geomorphology, i.e., Lesser, Sub and Higher Cholistan. Lesser Cholistan constitutes the western and north-western part of Bahawalpur dist. close to River Sutlej; flowing at the northern periphery of the study area (Fig 1). It is covered mainly with some vegetation over less fertile lands and sand cover as well [9–12]. Higher Cholistan is the eastern part that touches with international border with India and is predominantly covered with sand blankets and sand dunes. Sub Cholistan is the part between Lesser and Higher Cholistan. It is comprised of sand cover, sand dunes with low height and some vegetation cover. The height of sand dunes gradually increases from west to east, i.e., from Lesser to Higher Cholistan [5,13].

The study area lies in lesser Cholistan and River Sutlej flows almost parallel to the study area at its north. Whole of the Bahawalpur district lies in an arid region with intense summers and winters. River Sutlej is facing acute water shortage after Indus Water Treaty and climatic changes as well. However, the river course is somehow fed by an ample amount of water in flood seasons, so, it is still believed to be a primary source of recharging the underground alluvial sediments in Lesser Cholistan [8,14–16]. The recharge turns to minimal while going away from the river as the decrease in vegetation cover is evident of it. In Lesser Cholistan, the canal network also exists, feds the farming lands of the study area to some extent. The south and south-eastern part of the study area is generally comprised of sand-cover and sand dunes of low height. This sandy part is barren and almost devoid of vegetation and population as well. However, some small villages/tribes and nomadic communities live over there but the inhabitants suffer a lot to overcome their domestic needs by fetching water from far-off areas. Groundwater salinity can also be observed in several tobas and underground water wells in this area [17–21]. Generally, excessive withdrawal of good to better quality groundwater through water wells in upper reaches close to the River Sutlej, has also been observed in the last decades to fulfil the increasing agriculture and domestic demands in Lesser Cholistan. This activity also poses a threat of intermingling saline water into freshwater to recover the groundwater imbalanced concentration, consequently polluting the freshwater parts of the aquifer. In the Cholistan region, the groundwater potential and quality are generally poor, and hence its use is also limited [22–27].

Keeping in view the challenges of water scarcity and quality in Cholistan area, there is a dire need to cover a research gap of hydrogeophysical along with hydrogeological investigations to reassess the latest groundwater potential and delineation of different quality groundwater zones exist in the study area. The present study would be very helpful for the understanding of current groundwater conditions and its sustainability for the future. Moreover, the results of this research would also be a source of attention to the government and other stake-holders to device a long-term planning to secure the reserve of such a priceless nature’s gift.

Hydrogeophysical investigations were carried out in the study area by using geo-electrical survey. This survey is commonly known as the electrical resistivity survey (ERS) which is considered the best for exploitation of groundwater resources worldwide. It is a cost-effective, non-invasive and less time consuming approach of subsurface investigations [28–31]. Hydrogeological data in terms of borehole litho-logs and water table of the study area were analysed simultaneously with ERS results for final interpretation and validation of ERS results as well. Different qualities of aquifer pockets were evaluated in terms of electrical resistivity behaviour with depth. The hydrogeological values (Dar-Zarrouk parameters) were then estimated to demarcate the sharp boundaries among different qualities of groundwater zones. Finally, the hydraulic properties were estimated to evaluate the aquifer potential across the study area.

## Geological setup

**Fig 2:**
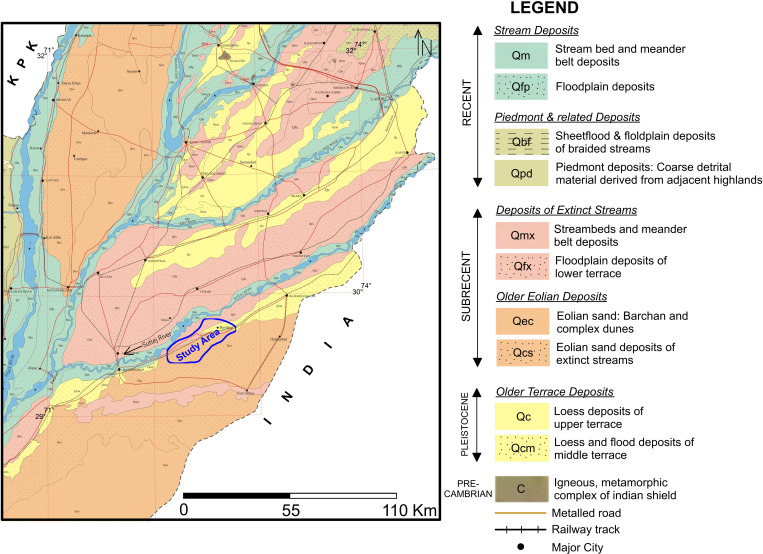
Geological map showing the surface geology of the study area and surroundings [38].

The study area is adjacent to the vast interfluves doabs system of Punjab Province and lies the east of the eastern river (Sutlej River) in Punjab Plain (Fig 1). Whole of the study area is comprised of flat land with gentle slope, i.e., less than one meter per km. Punjab Plain is also referred to as the Indus Plain as all the rivers flowing at the eastern side of the mighty Indus River are its tributaries. The geomorphological units of Punjab Plain area Central Alluvial Plain and Piedmont zone [5,17,32]. The subsurface alluvium deposition in the study area and in whole of the Cholistan is due to the current and paleo-channels of River Sutlej and the paleo-channels of Ghaggar-Hakra River system. This Quaternary alluvial cushion is believed to be in the continuity of sediments in the northern parts of the Province. However, these sediments are predominantly fine-textured as compared to that in the upper reaches of Indus River. Later on, in most part of Cholistan and the study area, this alluvial package was covered by eolian deposits of Holocene age comprising the existing sand-cover with sand dunes. The exploratory drilling works within and at peripheral parts of the study area endorse it. The drilling works also reveal that the alluvium succession overlies the sedimentary strata with fluviatile Siwaliks (Miocene-Pliocene age) at the top [10,11,33,34]. The sedimentary sequence overlies Precambrian basement rocks which are exposed as Indian Shield in the form of monadnocks near Chiniot, Sangla Hill, Sargodha and Shahkot areas in adjacent doabs at the north of the study area (Fig 2). Due to the flat lands with least orogeny and buried basement rocks, the Punjab Plain is also termed as Punjab Platform as well.

The alluvium (overlying the sedimentary strata) is composed of unconsolidated Quaternary (Pleistocene-Holocene) deposits primarily composed of intermixed sand, gravel, silt, clays and minor kankars [7,35–37]. The geology of the study area is characterized as “Floodplain deposits of lower terrace” especially close to Sutlej River, “Loess and flood deposits of middle terrace” and eolian sand with Barchan and complex dunes (Fig 2).

**Fig 3:**
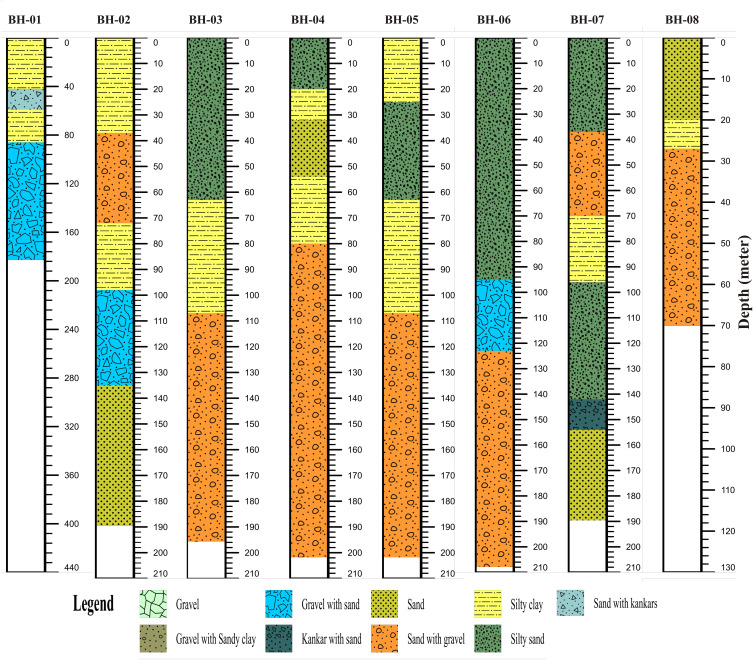
Litho-logs of boreholes drilled within the study area.

A careful appraisal of litho-logs of boreholes reveals that the Quaternary alluvium is about 200 meter in average thickness and along with fluviatile Siwaliks the thickness goes on 100’s of meter averaged. Groundwater reservoir/ aquifer in the study area and in doabs region are considered to exist in an unconfined state and are being abstracted from Quaternary alluvium [5,34,39,40]. The borehole lithological logs in the study area are shown in Fig 3 clearly indicating the major lithological units exist in the study area.

### Subsurface configuration of Punjab Platform

No part of the earth is without geology. Every part possesses its own geological details, whether exposed and visible or concealed and invisible. The exposed part can be studied directly whereas the concealed geology needs to be studied indirectly by means of geophysical tools or by drilling a hole to a certain depth [4,41–43]. A few decades back when it was talked about the geology of alluvium-covered areas of Punjab Platform, the researchers used to say that there is no significant geology in most of the parts of Punjab Platform. But gradually the activity of hydrocarbon exploration and other geophysical investigations gave some ideas about the nature of subsurface geology. Deep oil-wells litho-logs and wire-line logs have further improved this knowledge remarkably. On the basis of these oil-wells data and discrete seismic sections, many experts, familiar with the state-of-the-art, portrayed the subsurface geology of Indus Plain [36,44].

**Fig 4:**
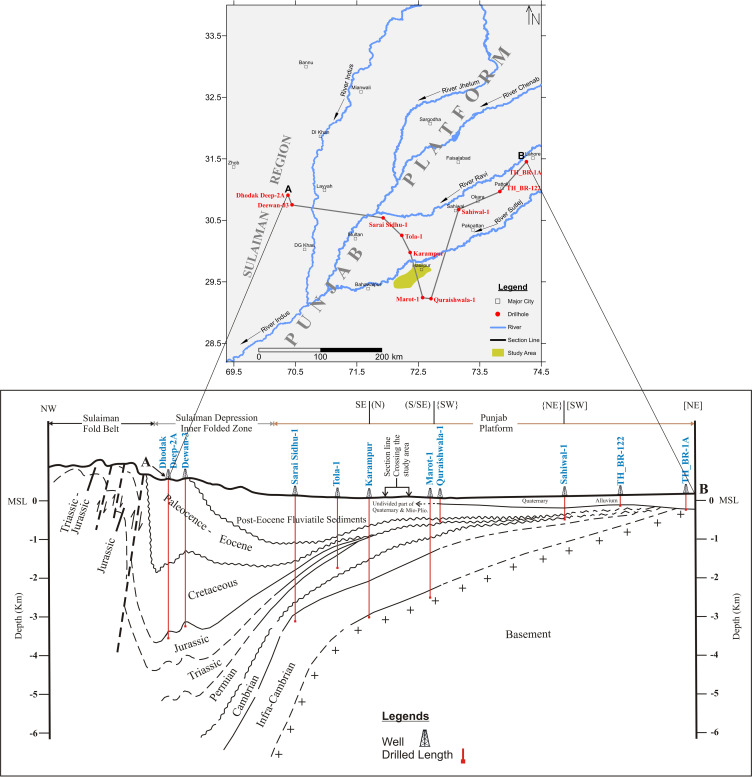
Regional subsurface model showing spatial variation of subsurface geology of Punjab Platform and adjacent Sulaiman region.

The volume of subsurface geological data has been increasing with time. In addition to deep wells of oil exploration companies, Geological Survey of Pakistan (GSP) has also carried out deep drilling at a number of places within Punjab Platform including Quraishwala, Minchinabad and Yousafwala (Sahiwal) in connection with coal exploration in Cholistan during 1993–99 [45–49]. Utilizing their boreholes data and other deep oil-wells data, we have further improved the schematic subsurface model (Fig 4) modified after Kadri (1995) [39].

Considering Fig 4, the continuous subsurface black lines represent the information with high accuracy extracted from real data of borehole lithological logs. While the discontinuous/dashed black lines show the inferred and extrapolated information due to the insufficiency of boreholes data. The actual drilling depths of boreholes from natural surface level (NSL) can also be observed. The model clearly deciphers that the basement goes deeper while moving towards the west and northwest and goes shallower towards the south, southeast and also northeast. The basement rises up to its shallowest position at dist. Lahore indicated by borehole TH_BR-1A where it lies right above the Quaternary alluvium. All the sedimentary sequence (Pliocene to Cambrian) pinches out and finally vanishes while approaching to Lahore region from the southwest. The model also reveals that the igneous-metamorphic basement complex underlying thick sedimentary sequence exists at about 3 km depth within the study area.

### Hydrogeological settings

The subsurface geology delineated through drilled holes indicates that the underlying Quaternary alluvium is almost pervious and holds water-bearing characteristics. Moreover, the coarser lithology of underlying Post-Eocene fluvial sediments holds almost the same characteristics too. The alluvium is mainly composed of relatively coarse grained sediments, i.e., sand and gravel, however, intermingling with fine sediments of clays and silts also exist trapping the groundwater from its free movement in underground water channels [7,39,40,50,51]. Boreholes data also indicates that the alluvium package goes to thicken towards the north and northeast where its thickness is about > 1000 ft. Generally, Punjab Platform holds a gigantic unconfined aquifer system fed primarily by Indus River along with its tributaries. The aquifer in the study area is also a part of it [12,34,52–54]. Clay and other fine materials also exist with intermixing of other coarser ones at different parts at subsurface levels. An increase in the amount of these finer materials within sub-layers tries to restrict groundwater flow and ultimately deteriorate the freshwater aquifer zones by increasing the salinity level. The areas close to the River Sutlej may have relatively better quality and quantity of groundwater, however, the situation gets worse while moving away from the river towards Higher Cholistan areas [21,55,56]. Overall the Cholistan area faces water scarcity and groundwater deterioration in terms of natural salinity which is a challenging issue for groundwater engineers and hydrogeologists.

### Dataset and methodology

The ERS is a highly reliable tool as compared to other hydrogeophysical investigations. It is being utilized worldwide to evaluate the electrical properties of subsoil materials that further lead to estimate the hydrogeological values which are ultimately used to get a detailed viewpoint of latest aquifer conditions [57–59]. A total of 50 evenly distributed ERS probes were selected for the best utilization of the present research work. A high-tech instrument was used for this purpose which records the electrical resistivity values at different subsurface levels in the field. During field data acquisition, the vertical electrical sounding (VES) technique using Schlumberger electrodes combination method was deployed as it is most effective approach for groundwater exploration especially in the alluvium strata. The following are the main technical key points of ERS;

- Electrical resistivity contrast should exist between the formations under study [60, 61].
- While carrying out the electrical resistivity survey using Schlumberger electrodes configuration, about two times the space along a straight line is required to achieve the estimated depth of investigation [31,62,63].
- Resistivity values of alluvial strata and bedrock in an area could be established if the subsurface lithology of at least one test hole or tubewell in or around that area is known to have similar geological conditions [64, 65].
- If the soil consists of thin alternate layers, the resistivity obtained at the surface would be the average effect of these alternate layers [37,57].

During the execution of the VES survey, apparent resistivity values are obtained for various depths below the surface by systematic expansion of current and potential electrodes from its center point along a straight line, while spacing between the electrodes is maintained. Moreover, the Schlumberger electrodes arrangement requires lesser electrode spacing on ground to achieve the required depth of investigation as compared to other types of electrodes arrangements. Lateral in-homogeneities can also be identified easily by using this arrangement [66–68]. A maximum of 200 m was the depth of investigation during VES survey of the present study area.

**Fig 5:**
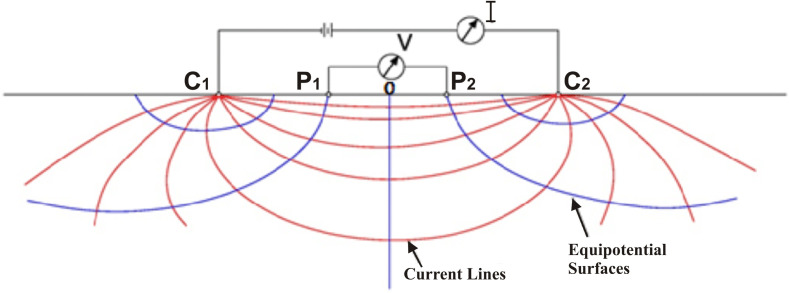
Conceptual diagram of electrical resistivity survey.


While taking measurements during the VES survey, commutated direct or low frequency current is introduced into the ground through a combination of current electrodes *C*_*1*_ and *C*_*2*_ fixed into the ground surface (Fig 5). Another pair of potential electrodes *P*_*1*_ and *P*_*2*_ is inserted in the ground between the outer current electrodes keeping in view that all electrodes should be collinear (Fig 5). Both the current and potential electrodes pairs are made up of stainless steel, however for measuring potential, porous pots can also be used instead of electrode rods [54,69,70]. The potential difference is measured between these two potential electrodes. By measuring current (*I*) flowing between two current electrodes and the associated potential difference (*V*) between the potential electrodes, the electrical resistivity (ρ) is computed by using the following Eq (1);

$$\rho =(K)*\left(\frac{V}{I}\right)$$ (1)

In the above equation, *K* represents the geometrical factor which is different for different electrode configurations. In homogeneous subsurface conditions, Eq (1) yields the true electrical resistivity of subsurface material. But in anisotropic and inhomogeneous conditions, it represents the weighted average electrical resistivity of the formations through which the current passes [68,70,71]. Since the subsoil is generally inhomogeneous and anisotropic, the resistivity value computed from Eq (1) is known as apparent resistivity denoted by $\rho_{a}$.

Therefore


$$\rho_{a}=(K)*\left(\frac{V}{I}\right)$$ (2)

During the data acquisition in the study area, half of the current electrodes spacing (*C*_*1*_ *C*_*2*_*/ 2*) was kept at 1–200 m, while half of the potential electrodes spacing was kept at 0.5 to 10 m. The electrodes spacing was increased gradually but in as systematic manner. The outer electrode spacing (*C*_*1*_ *C*_*2*_) actually corresponds to the depth of achievement. The apparent electrical resistivity values for different depth intervals were collected at a single VES probe using Schlumberger electrodes combination and the same was done for all probes. The coordinates of each VES probe were recorded through the Global Positioning System (GPS).

## Results and discussions

### Hydrogeophysical investigations

**Fig 6:**
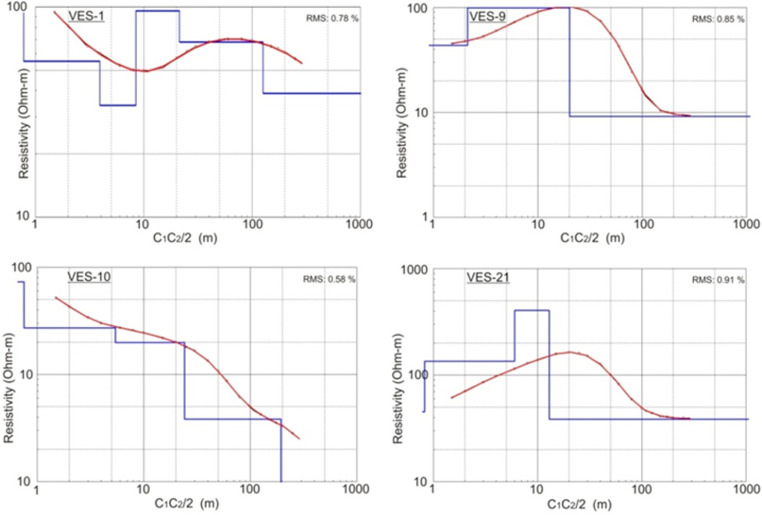
Processed models of some VES curves.

For the initial processing of field values of apparent resistivity, the partial curve matching method was used. For this purpose, Ebert auxiliary graphs were utilized [72]. However, the final processing was carried out by using computer-aided interactive software IPI2Win [73]. The automatic iteration was used in the software to develop the subsurface geo-electrical layered models for each VES probe. Afterwards the software processing, the true electrical resistivity values were generated in the form of curves/ graphs. Some of the processed curves are represented in Fig 6.

**Fig 7:**
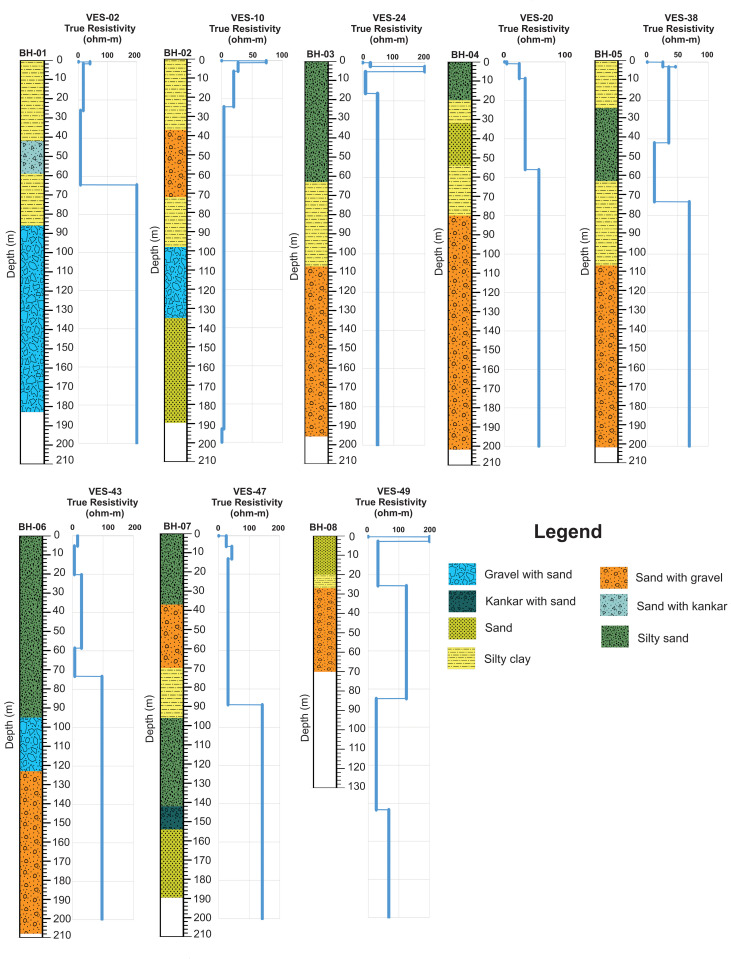
Calibration of lithological logs of boreholes with processed models of nearest VES probes.

Considering Fig 6, the apparent resistivity values (black circles) are at Y-axis and half of the current electrodes distances are at X-axis while logarithmic graphs are used. The red line represents the best-fit line with the line joining the tiny black circles (Fig 6). Finally, a blue line represents the processed model for each probe representing the thicknesses/ depths and processed resistivity values of each subsurface geo-electric layer (Fig 6). The processed resistivity values are also termed as true electrical resistivity values. In the study area, 3–6 geo-electric sub-layers were interpreted generally with specific thickness and true resistivity of each layer. The true resistivity values correspond to a particular lithology interpreted for specific geo-electrical layers. The range of processed true resistivity values is 1.98–200 Ω-m across the study area. After a careful analysis, resistivity values in the study area were categorized according to their interpreted lithology represented in Table 1. Moreover, the calibration of processed resistivity values of VES probes with subsurface lithology of nearest boreholes has its own importance in validating the VES data [51,54,74]. So, it was also done for the present study and represented in Fig 7. Water table assessment was also carried out across the study area during the VES survey through the available sources such as open wells, water pumps, lithological data of boreholes, tubewells etc. The water table varies about 10–24 m in the study area. To reach the final interpretation, several parameters were taken into account simultaneously such as processed VES data, field reconnaissance, lithological logs of boreholes, water table data, literature review and field checks of water samples as well.

**Table 1:** Customized resistivity ranges with cutt-off values and interpreted subsurface lithology.

Resistivity range	Resistivity cut-off values (Ω-m)	Subsurface interpreted lithology
High	>45–200	Intermixed layers with the predominance of sand-gravel with some admixture of interbedded fine silts/clays.
Medium	20–45	Intermixed layers with the predominance of silt and fine sand with some admixture of sandy sediments.
Low	<20	Intermixed layers with the predominance of fine material, i.e., clays/silts with minor/rare admixture of coarser sandy sediments.

**Fig 8:**
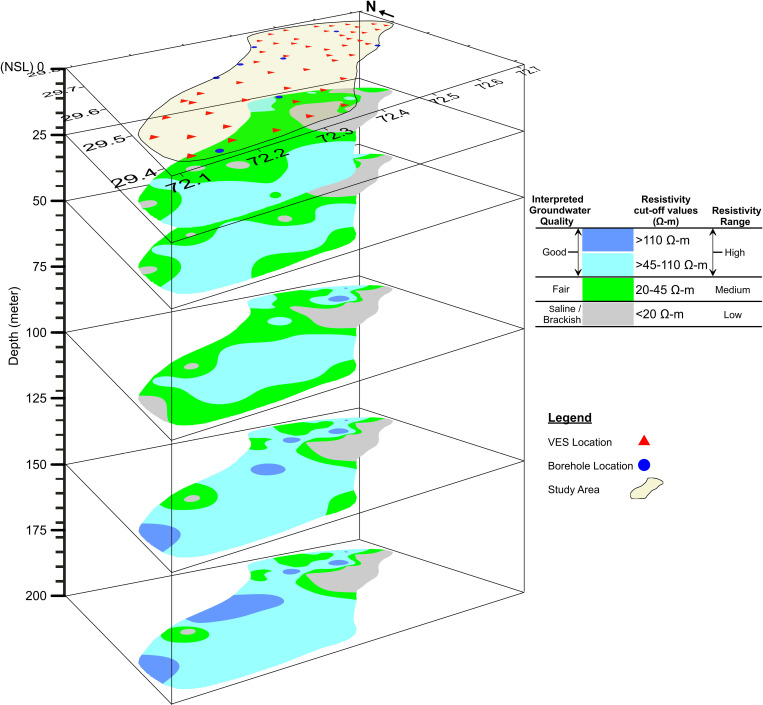
3D view showing different groundwater quality zones in terms of spatial variation of resistivity.

The resistivity values were further tabularized to develop contour maps at different depth levels with the aid of interactive software to observe the distribution of resistivity in the study area laterally and along with depth. These 2D maps further lead to delineate the subsurface groundwater conditions in terms of resistivity changes. All these maps were further stacked and developed a 3D perspective view showcasing the groundwater quality zones (Fig 8).

Fig 8 deciphers the groundwater conditions in terms of resistivity values below the water table. Grey color represents low resistivity zones (<20 Ω-m) and the lithology is interpreted to be composed of predominantly finer sediments such as intermixed clays and silts with a rare admixture of coarser ones, i.e., sand-gravel. These sediments are interpreted to be saturated with unfit (saline/brackish) groundwater quality below the water table conditions. Green color represents medium resistivity zones (20–45 Ω-m) which are believed to be associated with intermixed layers of silt with fine sand in dominance with a minor admixture of coarser sandy sediments. Below the water table, these sediments are interpreted to be saturated with marginal/fair quality groundwater. At last, both the shades of blue color (light and dark blue) represent high resistivity zones (>45–200 Ω-m) which are interpreted to be saturated with good quality groundwater under water table conditions within predominant coarser sediments such as intermixed sand-gravel. However, both shades differ due to the amount of gravel and kankar inclusions. The dark blue color represents comparatively more gravel and occasional kankars within sand dominant strata and due to this reason the resistivity values go on to the higher side, i.e., up to 200 Ω-m.

Considering Fig 8, some small patches of unfit groundwater condition (saline/brackish) appear close to the water table (showing with grey color). However, a comparatively large portion of the same groundwater condition becomes visible in the eastern and north-eastern part of the study area. This large saline portion persists with depth up to a maximum depth of achievement (200 m). A big portion of fair quality groundwater (showing with green color) also appears close to the water table but goes to shrink gradually with depth and good quality groundwater (showing with light and dark blue color) replaces it with depth. Comparatively small portions of dark blue color appear at 200 m depth representing the nominal existence of gravel/kankar inclusion within the coarser strata across the study area.

### Hydrogeological measurements

**Fig 9:**
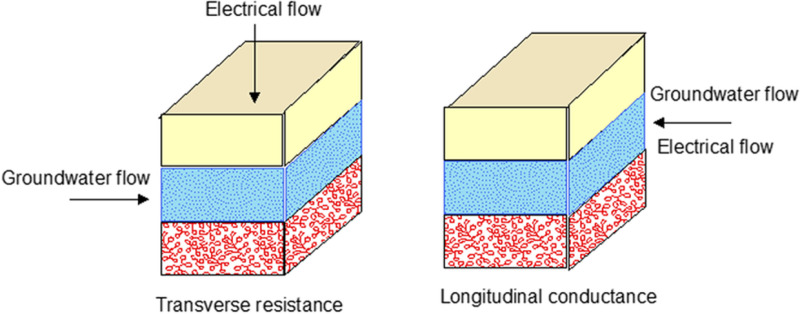
Conceptual model depicting transverse resistance and longitudinal conductance in subsurface strata.

When the electrical resistivity values of fine sediments (clay/silt) with coarser ones (sand) and saline water interface with each other, the interpretation turns into complexities. Such an issue requires a better formulation of interpretation to yield an understandable solution of differentiating fresh and saline/brackish groundwater zones of aquifer bodies [54,75,76]. Sometimes, it is also observed that the interpreted results of VES data exhibit intermingling of resistivity values caused by overlapping of fresh and saline/brackish groundwater zones in the aquifer. Consequently, the useful information merges and uncertainty prevails to some extent. Generally, the hydrogeological values in terms of Dar-Zarrouk (D-Z) parameters, evaluated from VES data, can avoid such kind of overlapping [6,77–79]. D-Z parameters include longitudinal conductance (*S*_*c*_), transverse resistance (*T*_*r*_) and longitudinal resistivity (*ρL*) can be computed through different arrangements/combinations of electrical resistivities and thicknesses of subsurface saturated layers in terms of geo-electric layers. Computing D-Z parameters gives an applicable solution to understand the geophysical signatures of different groundwater quality zones. *S*_*c*_ is the conductance parallel to the face and *T*_*r*_ is the resistance normal to the face for a unit cross-sectional subsurface area (Fig 9). These parameters play an essential role in resistivity soundings [76,80].

The calculations of D-Z parameters are made by the following Eqs (3)–(5).


$$S_{c}=h/\rho$$ (3)

$$T_{r}=\rho h$$ (4)

ρL=h/S (5)

**Fig 10:**
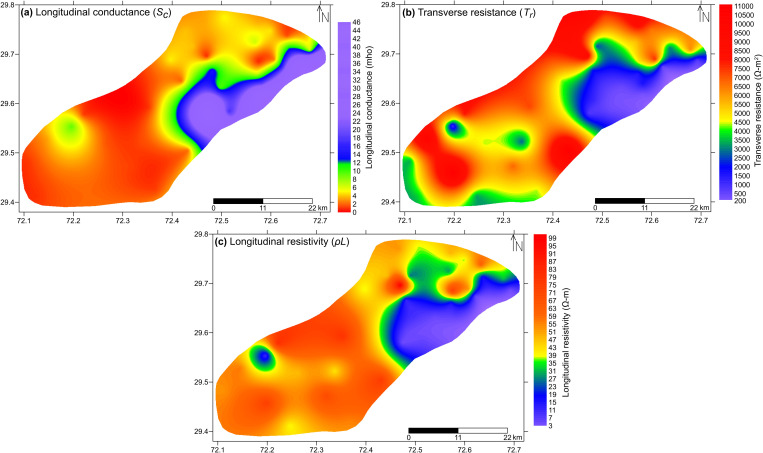
Distribution of D-Z parameters within the study area. (a) Longitudinal conductance, (b) transverse resistance and (c) longitudinal resistivity.

In the above equations, *S*_*c*_, *T*_*r*_ and ρL are expressed in mho, Ω-m^2^ and Ω-m respectively. Moreover, *h* and *ρ* represent the averaged values of thickness and electrical resistivity of subsurface saturated layers respectively and expressed in meters and Ω-m respectively. The processed VES models were used to extract *h* and *ρ* for each VES probe. With the help of these values, D-Z parameters (*S*_*c*_, *T*_*r*_, *ρL*) were computed by using Eqs (3)–(5). A specific range of D-Z parameters was obtained depending on the hydrogeophysical and boreholes data of the study area. The obtained values were further gridded and contoured to develop 2D maps by using interactive software to observe the spatial distribution of D-Z parameters across the study area (Fig 10).

The longitudinal conductance relates inversely with transverse resistance and longitudinal resistivity. Moreover, transverse resistance and longitudinal resistivity directly relates to each other. Fig 10 also exhibits the same behavior among these parameters. These D-Z parameters distribution maps decipher groundwater quality with more accurate demarcation of saline/brackish groundwater boundaries with the least overlapping of freshwater. The range of longitudinal conductance, transverse resistance and longitudinal resistivity is computed as 0.9–46 mho, 198–10846 Ω-m^2^ and 3–98.2 Ω-m respectively across the study area (Fig 10).

Considering Fig 10, the rising values of longitudinal conductance (≥12 mho) correspond to the zones of lower values of transverse resistance and longitudinal resistivity (≤2500 Ω-m^2^ and ≤25 Ω-m respectively). Such combinations of values are represented by violet and blue colors. Lithology in these zones is interpreted to be associated with predominantly fine sediments of clays and silts with minor intermixing of coarser material. These sub-layers are believed to be saturated with unfit (saline/brackish) groundwater quality. The zones of lower values of longitudinal conductance (<12 mho) appear as the zones of higher values of transverse resistance and longitudinal resistivity (>2500 Ω-m^2^ and >25 Ω-m respectively). The combinations of these values are represented by other than violet and blue colors. These values represents relatively coarser lithology gradually increasing in grain size, i.e., silt with fine sand towards sand-gravel in dominance. The sediments layers are believed to be saturated with fair to good quality groundwater.

The yield of an aquifer through the pumping well depends upon two foremost properties; capacity of storage and capability to transmit groundwater. The hydrogeological values such as transmissivity and hydraulic conductivity represent these two vital aquifers properties. These values are commonly described as hydraulic properties of aquifers as they represent the hydraulic behavior of the saturated strata. In the groundwater management sciences, the hydraulic properties of aquifer are essential aiding to determine groundwater potential in different parts of an area under study. The researchers introduced many techniques to find out these properties for porous medium. Generally, these parameters are calculated by using pumping test data [39,51,81,82]. However, many attempts have also been introduced by different workers to evaluate these parameters through electrical resistivity data where the pumping test data is insufficient or even unavailable. Basically, the hydraulic and electric properties of subsurface strata directly correlate with each other because both of these are the function of heterogeneity, pore-spaces and size/ structure of grains [79,83–85].

The procedure of pumping test is expensive, time consuming and requires labors and instrumentations. On the other side, the hydrogeophysical technique is relatively cheaper and non-invasive. Usually, it is also impossible to allocate high budget for drilling the test wells to execute pumping test in an area under study. VES method is a type of hydrogeophysical technique that gives an effective, cheap and quick alternate route to estimate such importance hydrogeological information with the help of its quantitative evaluation technique [6,86–88].

Transverse resistance (one of the D-Z parameters) is proportional to the transmissivity and hydraulic conductivity is related to the transmissivity [53,81,86,88,89]. So, the hydraulic properties can be calculated by using Eqs (6), (7).


$$T_{r}=(0.19T^{\left(1.28\right)}$$ (6)

K=T/b (7)

**Fig 11:**
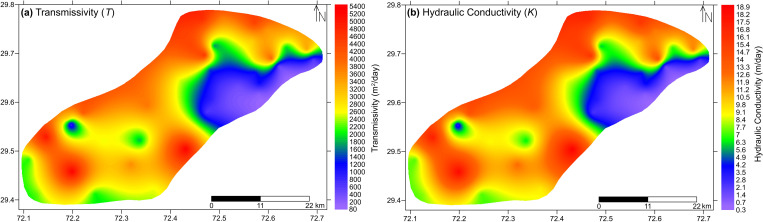
Distribution of hydraulic parameters within the study area (a) transmissivity and (b) hydraulic conductivity.

The symbols *T* and *K* represent transmissivity and hydraulic conductivity in m^2^/day and m/day respectively. *T*_*r*_ represents transverse resistance in Ω-m^2^ and *b* is the aquifer thickness in meter. By using Eqs (6), (7), *T* and *K* at each VES location were computed which further developed in 2D representation to observe the spatial distribution of these parameters (Fig 11).

Fig 11 represents the transmissivity and hydraulic conductivity values across the study area in the form of colors variation with a range of 80–5300 m^2^/day and 0.3–19 m/day respectively. Considering Fig 11, the zones of low transmissivity (≤1400 m^2^/day) and low hydraulic conductivity (≤6 m/day) are represented by violet and blue colors respectively and interpreted to be associated with the predominance of fine materials such as silts and clays saturated with unfit (saline/brackish) groundwater quality. Green color of transmissivity (1400–2500 m^2^/day) and hydraulic conductivity (6–9 m/day) showcase medium values which represent silt and fine sand in dominance and saturated with fair groundwater quality. Yellow color merging into red represents progressively increasing values and is categorized as high values of transmissivity and hydraulic conductivity (>2500 m^2^/day and >9 m/day respectively). These values represent sand-gravel in dominance with saturation of good groundwater quality under water table conditions.

**Fig 12:**
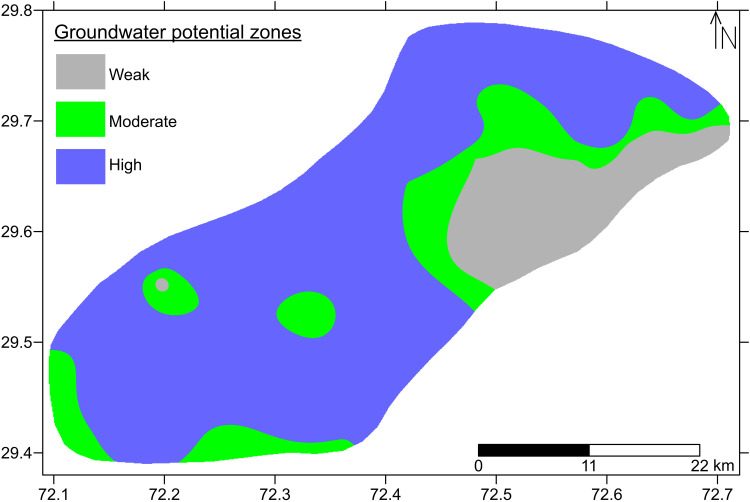
Groundwater potential zones across the study area.

The transmissivity correspond to the groundwater potential which ultimately reflects the aquifer yield. Grouping all of the high, medium and low transmissivity values, represents different groundwater potential zones across the study area. Fig 12 showcases different groundwater potential zones that exist in the study area. Grey, green and blue colors represent low, medium and high transmissivity respectively which eventually reflect the zones of relatively weak, moderate and high groundwater potential respectively. High and low potential zones confirm the occurrence of coarser and finer sediments in dominance respectively as a result of VES and D-Z parameters. Moderate potential refers to the lithology of progressively increasing grain size, i.e., silt and fine sand in dominance which is also in accordance with results of VES, D-Z parameters. Fine lithology affects the aquifer yield while coarser lithology provides ease for transmission of groundwater in underground channels. Almost 70% of the study area holds high groundwater potential while approx. 20% holds weak groundwater potential. Some small patches also exists carries moderate potential. The results of VES, D-Z parameters and hydraulic parameters are in accordance and support each other.

## Conclusions

Hydrogeophysical allied with hydrogeological approaches were utilized for the appraisal of current groundwater conditions in the northeast part of Bahawalpur district; a part of Lesser Cholistan, Punjab Province of Pakistan. Based on hydrogeophysical investigations, 3–6 geo-electric sub-layers were identified, however, their thicknesses and depths vary across the study area. The subsurface alluvium package is comprised of intermixed layers of sand, clay, silt, gravel and minor kankars. The resistivity values are categorized as low, medium and high in the study area. Different qualities of groundwater zones are delineated in terms of electrical resistivity behavior. Low resistivity zones (<20 Ω-m) are associated with predominantly fine sediments such as intermixed clays and silts. These sediments are interpreted to be saturated with unfit (saline/ brackish) groundwater quality below the water table. Medium resistivity zones (20–45 Ω-m) are believed to be associated with intermixed layers of silt with fine sand predominantly. Below the water table conditions, these sediments are interpreted to be saturated with fair groundwater quality. High resistivity zones (>45–200 Ω-m) are interpreted to be saturated with good quality groundwater under water table conditions within predominantly coarser sediments of intermixed sand-gravel. The higher resistivity values >110 Ω-m refers to the increasing amount of gravel within coarser strata. Almost 70% of the study area reflects coarser lithology saturated with good groundwater quality.

More precisely demarcation of saline/brackish groundwater pockets has also been done by computing the hydrogeological values. High longitudinal conductance (≥12 mho) with low transverse resistance (≤2500 Ω-m^2^) and low longitudinal resistivity (≤25 Ω-m) represent predominantly fine sediments of clays and silts saturated with unfit (saline/brackish) groundwater quality. Low longitudinal conductance (< 12 mho) with high transverse resistance (>2500 Ω-m^2^) and high longitudinal resistivity (>25 Ω-m) represent relatively coarser lithology gradually increasing in grain size, i.e., silt with fine sand towards sand-gravel in dominance. The sediments are interpreted to be associated with fair to good quality of groundwater respectively.

Comparatively low values of hydraulic parameters (transmissivity ≤ 1400 m^2^/day and hydraulic conductivity ≤ 6 m/day) are delineated in the study area. These low hydraulic zones are interpreted as the strata of predominant fine materials such as silts and clays saturated with unfit (saline/brackish) groundwater. Medium hydraulic parameters (transmissivity 1400–2500 m^2^/day and hydraulic conductivity 6–9 m/day) correspond to silt and fine sand in dominance, saturated with fair groundwater quality. High hydraulic parameters (transmissivity >2500 m^2^/day and hydraulic conductivity >9 m/day) represent sand-gravel in dominance with the saturation of good groundwater quality under water table conditions.

Almost 70% of the study area holds high groundwater potential represents coarser lithology in dominance which provides an ease to transmit water in subsurface channels that ultimately leads to a water well of good yielding capacity. About 20% of the study area especially the northeast part holds weak groundwater potential which represents fine lithology in dominance. These fine sediments affect groundwater transmission which eventually tries to weaken its potential. Some of the patches adjacent to low potential zones also exist, carrying moderate groundwater potential which reflects the increasing grain size compared with the lithology of weak groundwater potential.

## Supporting information

S1 File Data of true resistivity at different depth levels. (XLS)

S2 File Data sheet of aquifer’s parameters estimation. (XLS)

S3 File Coordinates of boreholes in the study area. (XLSX)

S4 File Water table data. (XLSX)
